# Diagnostic yield of inpatient capsule endoscopy

**DOI:** 10.1186/s12876-022-02323-9

**Published:** 2022-05-12

**Authors:** Irving Levine, Soonwook Hong, Dimpal Bhakta, Matthew B. McNeill, Seth A. Gross, Melissa Latorre

**Affiliations:** 1 Division of Gastroenterology, Department of Medicine, Northwell Health, New York, NY USA; 2 Department of Medicine, Beth Israel Deaconess Medical Center, Boston, MA USA; 3 Division of Gastroenterology, Hepatology and Nutrition, UTHealth McGovern Medical School, Houston, USA; 4 Division of Gastroenterology and Hepatology, New York University Langone Health, New York, NY USA

**Keywords:** Capsule endoscopy, Inpatient, Obscure gastrointestinal bleeding, GI hospitalist, Double balloon enteroscopy, Deep enteroscopy, Push enteroscopy, GI bleeding

## Abstract

**Background:**

Capsule endoscopy (CE) provides a novel approach to evaluate obscure gastrointestinal bleeding. Yet CE is not routinely utilized in the inpatient setting for a variety of reasons. We sought to identify factors that predict complete CE and diagnostically meaningful CE, as well as assess the impact of inpatient CE on further hospital management.1 na d2

**Methods:**

We conducted a retrospective review of patients undergoing inpatient CE at a tertiary referral, academic center over a 3 year period. We analyzed data on patient demographics, medical history, endoscopic procedures, hospital course, and results of CE. The primary outcome was complete CE and the secondary outcome was positive findings of pathology on CE.

**Results:**

131 patients were included (56.5% were men 43.5% women, median age of 71.0 years). Overall, CE was complete in 77.1% of patients. Complete CE was not related to motility risk factors, gender, or administration modality. Patients with incomplete CE tended to be older, have lower BMI, and Caucasian, however results did not reach statistical significance (*p* = 0.06; *p* = 0.06; *p* = 0.08 respectively). Positive CE was noted in 73.3% of patients, with 35.1% of all patients having active bleeding. Positive CE was not associated with AVM risk factors or medication use. 28.0% of patients underwent subsequent hospital procedures, among which 67.6% identified the same pathology seen on CE.

**Conclusions:**

Contrary to previous studies, we found the majority of inpatient CEs were complete and positive for pathology. We found high rates of correlation between CE and subsequent procedures. The use of CE in the inpatient setting helps to guide the diagnosis and treatment of hospitalized patients with obscure gastrointestinal bleeding.

## Introduction

Video capsule endoscopy (CE) provides a novel non-invasive method to evaluate patients with obscure gastrointestinal bleeding. After initial endoscopy and colonoscopy, clinicians often struggle to determine the next appropriate diagnostic modality, including repeat endoscopy, radiographic modalities, or small bowel evaluation with push enteroscopy or balloon-assisted deep enteroscopy. In contrast to the above methods, CE provides a non-invasive modality to directly visualize the small bowel in its entirety. CE aids in the diagnosis and management of several small bowel diseases, including but not limited to inflammatory bowel disease, celiac disease, polyposis, and gastrointestinal bleeding [1–3].

In patients with suspected small intestinal bleeding, CE has demonstrated superiority in identifying sources of bleeding in comparison to both push enteroscopy and small bowel barium radiography [4, 5]. In addition to the high rates of meaningful diagnostic findings, there are few contraindications to CE, unlike the other modalities [6]. As such, current European Society of Gastrointestinal Endoscopy (ESGE) and American College of Gastroenterology (ACG) guidelines recommend CE as first-line investigation for patients with obscure gastrointestinal bleeding [7, 8].

Despite these advantages, CE is not routinely used in the inpatient setting for several reasons. First, variable reimbursement and cost of equipment often precludes inpatient CE. Additionally, prior studies have demonstrated decreased completion rates among inpatient CE [9, 10]. Hospitalized patients tend to be more sedentary and are prone to delayed transit time through the gastrointestinal tract [9]. However, while previous studies have noted the lower completion rates of CE among inpatients, they have failed to characterize patient and clinical factors that predict these incomplete CE.

Moreover, the diagnostic potential of CE carries tremendous importance. Patients with obscure gastrointestinal bleeding tend to have prolonged length of stay, frequent hospital admissions, and invasive procedures [11]. Determining an etiology of obscure gastrointestinal bleeding has the potential to decrease length of stay, prevent readmissions, and prevent repeat unnecessary invasive procedures.

Lastly, to our knowledge, previous studies have failed to correlate the results of inpatient CE and further inpatient management. CE has the potential to greatly impact the selection of further therapeutic interventions which is a decision that often challenges clinicians. CE can help differentiate the patients who would most benefit from further endoscopic procedures (or push enteroscopy or balloon-assisted deep enteroscopy), surgical intervention, medical management, or even no further intervention at all.

In January 2018 NYU Langone Health obtained the necessary technology to perform small bowel video capsule endoscopy for hospitalized patients onsite. The hospital had also recently transitioned to a gastroenterology (GI) hospitalist led consult service which established the in-house availability of a gastroenterologist trained in video capsule endoscopy and balloon-assisted deep enteroscopy. Thereafter small bowel evaluation with video capsule endoscopy became a regularly utilized resource in the evaluation of obscure gastrointestinal bleeding for hospitalized patients in accordance with recent guidelines [7, 8].

In this study, we sought to (1) identify patient and clinical factors that predict complete inpatient CE; (2) identify predictors of yielding diagnostic results on inpatient CE; and (3) assess impact of inpatient CE on further hospital management and rates of 30-day readmission.

## Methods

### Study population and variables

We identified all patients age 18 and older undergoing inpatient CE at NYU Langone Health Tisch Hospital/Kimmel Pavilion between January 1, 2018 and October 30, 2019. Charts were reviewed for patient demographic information, including age, gender, and medical history. Elderly status was defined as age over 65 years at the time of CE. Obscure gastrointestinal bleeding was defined as gastrointestinal bleeding without a known source despite esophagogastroduodenoscopy (EGD) and colonoscopy. Indication for CE was divided into overt obscure gastrointestinal bleeding (hematemesis, hematochezia, or melena) or occult obscure gastrointestinal bleeding (iron-deficiency anemia, chronic anemia). Motility risk factors were defined as comorbid diabetes mellitus or systemic disease affecting motility (i.e. scleroderma, hypothyroidism, amyloid). Arteriovenous malformation (AVM) risk factors were defined as having comorbid cardiac valvular disease or advanced kidney disease (CKD stage 3–5). Charts were reviewed for hospital course, including length of stay, laboratory findings, radiographic imaging, procedures, and rates of readmission. Thirty-day readmission was defined as non-elective hospital admission at NYU Langone Health within 30-days of discharge from index admission.

### Capsule endoscopy protocol

CE with PillCam SB3 12 h capsule was used. All patients who underwent CE had either nothing by mouth (except for medication) during the 12 h before CE administration or had a bowel preparation (2 L polyethylene glycol or equivalent preparation, or 4 L if performed after colonoscopy preparation). CE was administered either orally or endoscopically, at the discretion of the administering gastroenterologist.

### Capsule endoscopy results

All CE were reviewed by the same GI hospitalist to ensure consistency. CE were defined as complete (complete CE) if the capsule passed into the cecum within the recording period. The yield of diagnostically meaningful pathology on CE (positive CE) was defined as finding abnormal small bowel bleeding pathology. Abnormal small bowel bleeding pathology was characterized as arteriovenous malformation (AVM), small bowel ulceration or erosion, small or old blood in the lumen with no identifiable source, brisk active bleeding, gastritis/duodenitis, or polyp/mass. Additionally, the presence or absence of active bleeding was identified.

### Subsequent procedures

Charts were reviewed for subsequent procedures following inpatient CE during the index admission. Subsequent procedures included EGD, colonoscopy, surgery, interventional radiology procedure, double balloon enteroscopy (antegrade or retrograde), and push enteroscopy. All subsequent procedures operative notes and images were reviewed by a single gastroenterologist to determine if the findings correlated with CE findings.

The primary outcome was complete CE. Secondary outcome was positive CE.

### Statistical analysis

Continuous variables were summarized using means and standard deviations and compared using the Student’s t-test, whereas categorical variables were summarized using proportions and compared using Chi-square test. All tests were considered significant at a 2-sided *p*-value less than 0.05. STATA was used to perform all statistical analyses. The study was approved by the NYU Langone Health Institutional Review Board.

## Results

### Study population

A total of 131 patients were identified. Overall, the median age was 71.0 years, 62.6% were elderly, 56.5% male and 43.5% female (Table 1). 87.0% of patients received bowel preparation (2–4 L of polyethylene glycol preparation solution or equivalent) prior to the CE. Motility risk factors were identified in 49.6% of patients. AVM risk factors were identified in 37.4% of patients. The most common indication for CE was overt obscure bleeding (n = 81; 61.8%) followed by occult obscure bleeding (n = 48; 36.6%).

**Table 1:** Baseline demographics and complete capsule endoscopy

Characteristic, n (%)	Total	Complete CE (n = 101; 77.1%)	Incomplete CE (n = 30)	*p*-value
Age, years (median, IQR)	71.0, 19.0	69.0, 19.5	75.0, 18.8	*p* = 0.06
Elderly (Age ≥ 65)	82 (62.6%)	59 (58.4%)	23 (76.7%)	*p* = 0.08
Male	74 (56.5%)	58 (57.4%)	16 (53.3%)	*p* = 0.69
Female	57 (43.5%)	43 (42.6%)	14 (46.7%)	
BMI (median, IQR)	26.1	23.8	27.0	*p* = 0.06
Obesity (Y/N)	40 (30.5%)	35 (34.7%)	5 (16.7%)	*p* = 0.08
Caucasian	81 (62.3%)	58 (57.4%)	23 (79.3%)	*p* = 0.08
Bowel prep	114 (87.0%)	86 (85.1%)	28 (93.3%)	*p* = 0.26
Motility risk factors	65 (49.6%)	54 (53.5%)	11 (36.7%)	*p* = 0.11
DM	54 (41.5%)	45 (45.0%)	9 (30.0%)	*p* = 0.15
Opioids	9 (6.9%)	8 (7.9%)	1 (3.3%)	*p* = 0.40
Oral administration	99 (75.6%)	76 (75.3%)	23 (76.7%)	*p* = 0.87
LOS (median, IQR)	6.0, 7.0	5.0, 6.0	6.0, 9.3	*p* = 0.32
Admitted 30-d before index admission	33 (25.2%)	26 (25.7%)	7 (23.3%)	*p* = 0.79
Readmitted 30-d after index admission	25 (19.1%)	21 (20.8%)	4 (13.3%)	*p* = 0.37
SBTT, min. (median, IQR)		265.0 (195.5)	N/A	N/A

*BMI* Body Mass Index; *DM* Diabetes Mellitus; *LOS* Length of Stay; *SBTT* Small Bowel Transit Time

### Complete CE

101 patients (77.1%) had complete CE. Complete CE was not related to gender, mode of capsule administration, or motility risk factors. (Table 1) Patients with incomplete CE tended to be older (75 years vs 69 years), elderly (76.7% vs 58.4%), have a lower BMI (23.8 vs 27.0), and Caucasian (79.3% vs 57.4%), however these did not reach statistical significance (*p* = 0.06; *p* = 0.08; *p *= 0.06; *p* = 0.08 respectively). CE completion was not associated with LOS or 30-day readmission rates.

### Positive CE

96 patients (73.3%) had a positive CE. (Table 2) Positive CE was not associated with AVM risk factors or NSAID use. Use of PPI, anticoagulation or antiplatelet were not associated with positive CE. BMI tended to be higher in patients with complete CE and those with positive CE, however this did not reach statistical significance. (Table 3).

**Table 2:** Rates of meaningful diagnostic yield on CE

Characteristic, n (%)	Total	Diagnostically meaningful pathology on CE (n = 96;73.3%))	No diagnostically meaningful pathology on CE (n = 35)	*p*-value
Age (median, IQR)	71.0, 19.0	71.5, 17.5	69.0, 35.0	*p* = 0.10
Male	74 (56.5%)	56 (58.3%)	18 (51.4%)	*p* = 0.48
Female	57 (43.5%)	40 (41.7%)	17 (48.6%)	
AVM risk factors	49 (37.4%)	39 (40.6%)	10 (28.6%)	*p* = 0.21
NSAID	18(13.7%)	11 (11.5%)	7 (20.0%)	*p* = 0.21
PPI	58 (44.3%)	47 (49.0%)	11 (32.4%)	*p* = 0.07
Anticoagulation	39 (29.8%)	31 (32.3%)	8 (22.9%)	*p* = 0.30
Antiplatelet	56 (42.8%)	42 (43.8%)	14 (40.0%)	*p* = 0.70
Bowel preparation	114 (87.0%)	82 (85.4%)	32 (91.4%)	*p* = 0.37
Presenting Hg (median, IQR)	7.4 (3.0)	7.4 (3.1)	7.8 (3.0)	*p* = 0.58
LOS (median, IQR)	6.0 (7.0)	6.0 (7.0)	5.0 (6.0)	*p* = 0.32
Admitted 30-d before index admission	33 (25.2%)	26 (27.1%)	7 (20.0%)	*p* = 0.41
Readmitted 30d after index admission	25 (19.1%)	21 (21.9%)	4 (11.4%)	*p* = 0.19
Active bleeding on CE		46 (35.1%)	N/A	N/A
Elderly	82 (62.6%)	62 (64.6%)	20 (57.1%)	*p* = 0.44

*AVM* Arteriovenous Malformation; *PPI* Proton Pump Inhibitor; *Hg* Hemoglobin; *LOS* Length of Stay

**Table 3:** Association of BMI and complete CE or positive CE

Characteristic, N(%)	Total	Complete CE	Incomplete CE	*p*-value	Positive CE	Negative CE	*p*-value
BMI (median, IQR)	26.1	27	23.8	*p* = 0.06	26.3	24.7	*p* = 0.43
Normal weight (BMI < 25)	53 (40.5%)	36 (35.6%)	17 (56.7%)	*p* = 0.08	36 (37.5%)	17 (48.6%)	*p* = 0.93
Overweight or obese (BMI > 25)							
Overweight (BMI 25–29.9)	36 (27.5%)	29 (28.7%)	7 (23.3%)		30 (31.3%)	6 (17.1%)	
Obesity Class 1 (BMI 30–34.9)	21 (16.0%)	19 (18.8%)	2 (6.7%)		16 (16.7%)	5 (14.2%)	
Obesity class 2 (BMI 35–39.9)	8 (6.1%)	5 (5%)	3 (10%)		5 (5.2%)	3 (8.6%)	
Obesity class 3 (BMI > 40)	12 (9.2%)	11 (10.9%)	1 (3.3%)		9 (9.4%)	3 (8.6%)	

A total of 46 patients (35.1%) were found to have active bleeding. The most common finding among positive CE was AVM (n = 46; 47.9% of positive CE, Table 4), followed by brisk active bleeding (n = 25; 26.0%).

**Table 4:** Specific CE findings among patients with positive CE

Specific findings	n	% of total CE	% of positive CE
Active bleeding (y/n)	46	35.1	47.9
AVM	46	35.1	47.9
SB ulceration/erosion	11	8.4	11.5
slow/old/small blood in lumen, no source	23	17.6	24.0
Brisk active bleeding	25	19.0	26.0
Gastritis/duodenitis	14	10.7	14.6
Polyp/mass	6	4.6	6.3

*AVM* Arteriovenous Malformation; *SB* Small Bowel

### Subsequent hospital procedures

A total of 37 patients (27.5%) underwent subsequent hospital procedures for the same indication. Several patients underwent multiple subsequent procedures, for a total of 45 subsequent procedures. (Table 5) The most common subsequent procedure was double balloon enteroscopy (n = 24; 53.3% of subsequent procedures) followed by push enteroscopy (n = 12, 26.7% of subsequent procedures). Among the 37 patients who underwent subsequent procedures, 25 (67.6%) identified the same pathology as seen on CE.

**Table 5:** Subsequent procedures following inpatient CE

Subsequent procedure	n	% of subsequent procedures	% of total CE	% of positive CE
Capsules that lead to subsequent procedures	37	N/A	28.0	38.5
Total procedures	45			
Double balloon enteroscopy	24	53.3%	18.3	25.0
Push enteroscopy	12	26.7%	9.2	12.5
IR procedure	1	2.2%	0.8	1.0
Surgery	0	0.0%	0.0	0.0
Colonoscopy	5	11.1%	3.8	5.2
EGD	3	6.7%	2.3	3.1

*IR* Interventional Radiology; *EGD* esophagogastroduodenoscopy

### Readmission rates

A total of 33 (25.2%) of patients were admitted within 30-days prior to index admission. Additionally, 25 (19.1%) of patients were readmitted within 30-days after index admission. Neither prior admission nor subsequent readmission were associated with complete CE or positive CE.

## Discussion

In this retrospective study of patients undergoing inpatient CE, we found high rates of complete and positive CE. The clinical significance of these findings suggests a prominent role of inpatient CE in identifying and diagnosing the etiology of obscure gastrointestinal bleeding. Furthermore, we found congruence between CE results and subsequent endoscopic procedures, suggesting CE can assist in the selection of appropriate subsequent procedures and therapeutic intervention. To our knowledge, our study is the first to correlate CE with subsequent inpatient procedures.

Our finding of a 77.1% completion rate is higher than prior inpatient studies and comparable to outpatient studies [9, 10, 12–14]. Ben-Soussan et al. [15] first identified inpatient status as a risk factor for gastric capsule retention among 29 inpatients. Subsequent studies have noted inpatient status as a risk factor for incomplete small bowel evaluation with CE, noting completion rates as low as 65–69% [9, 10, 16]. The proposed mechanism for decreased completion rates among hospitalized patients includes immobility, the supine position of most hospitalized patients, stress induced by acute illness, and gastroparesis, either attributed to an underlying medical condition (i.e. diabetes mellitus, hypothyroidism, or scleroderma) or medication use [9, 15]. Yet all of these studies were limited by small numbers of inpatients, ranging from 29 to 70 patients. Our higher completion rate may relate to our standardized protocol for bowel preparation prior to CE, as 85.1% of patients had polyethylene glycol before the CE.

Our study found several patient demographics that trended towards incomplete CE, including older age, elderly status, lower BMI, and Caucasian race, yet these did not reach statistical significance. It is particularly noteworthy that lower BMI, and lack of obesity, were associated with incomplete CE, as obesity is correlated with gastroparesis. Larger studies are necessary to further evaluate these characteristics.

The lack of association between mode of capsule administration and completion rate is noteworthy, as delayed gastric transit time has been associated with incomplete CE [10, 15]. As such, one may hypothesize that bypassing the stomach by administering the capsule endoscopically into the duodenum may lead to higher rates of complete CE. This may have perhaps been mediated by the use of 12 h capsules. Larger trials are necessary to evaluate this hypothesis further.

Our findings of positive CE fall within the range of reported positive CE in outpatient studies (38% and 83%) [17–22]. Among the few inpatient studies, a recent retrospective study of 204 patients (103 inpatients) found the diagnostic yield of identifying a bleeding source by CE was 39% among inpatient compared to 34% outpatient (findings not statistically significant) [23]. When performed for obscure bleeding with reported overt blood loss, they found a significant difference (Diagnostic yield: 49% inpatient vs 23% outpatient; OR 3.32, 95% CI 1.25–8.84, *p* = 0.02). Other retrospective studies have found that advanced age was associated with increased diagnostic yield of CE [24–26]. We observed a trend towards elderly age being associated with a positive result on capsule endoscopy which did not reach statistical significance. We propose our high rates of positive CE may relate to proper selection of patients, as the majority of patients had previous endoscopy and/or colonoscopy prior to CE. Utilizing CE in inpatient settings for its diagnostic potential is imperative. By establishing a diagnosis, patients are spared from unnecessary repeat invasive procedures on subsequent hospitalizations, thereby increasing patient safety and decreasing hospital costs.

Of note, prolonged small bowel transit time, which may lead to incomplete CE, has been associated with higher diagnostic yield [27–29] As such, the endpoints of complete CE and positive CE may have an inverse relationship.

Identifying the role of inpatient CE in guiding further management remains a major factor preventing its widespread use. We identified that a large proportion of our patients underwent a subsequent inpatient procedure following CE for the same indication. Within the limitations of a retrospective study, we assume that the selection of which subsequent procedure was directly influenced by the CE results, and the high rate of congruence between CE findings and invasive procedure findings attests to this. As such, inpatient CE not only provides diagnosis, but can also aid in therapeutic intervention. Utilizing inpatient CE results to perform subsequent procedures in a timely fashion during the same hospitalization provides therapeutic intervention, can potentially decrease length of stay and hospital readmissions, and can certainly decrease delays that would take place in the outpatient setting. In particular, the current study benefited from being performed in a tertiary academic center where double balloon enteroscopy is routinely performed and a GI hospitalist is available on-site to accommodate the procedure in a timely manner. It is possible that patients in similar hospital systems will see the greatest benefit from inpatient CE, as the majority of subsequent procedures performed in the current study were double balloon enteroscopy.

Patients with obscure gastrointestinal bleeding have frequent bleeding events, often requiring hospitalization. Our study is the first to our knowledge to analyze readmission after inpatient CE. Our rates of 30-day readmission is higher than other studies assessing admission rates for obscure gastrointestinal bleeding following double balloon enteroscopy [30]. We anticipated that CE would affect hospital readmission in two opposite manners. First, several investigators have noted negative CE predicts low rebleeding risk, though this conclusion is contested by other studies [31, 32]. Alternatively, specific treatment after positive CE is associated with decreased rebleeding [32]. By identifying culprit lesions and providing therapeutic interventions, we anticipated positive CE findings would be associated with decreased hospital readmission. However, positive CE was not associated with decreased 30-day readmission. We suspect that 30-day follow up is too short an interval to assess for decreased hospitalizations, as previous studies have noted median time to rebleeding is 15 months [33]. Therefore, longer follow-up time is necessary to assess whether inpatient CE can decrease hospital admissions.

The strengths of the current study include the large cohort of inpatient CE. Additionally, it is the first study to correlate CE with subsequent endoscopic procedures, as well as assess readmission after inpatient CE. There are several limitations of our study. The retrospective nature precludes controlling for all cofounders, and relies on information documented in the electronic medical record. We also recognize the potential lack of generalizability as the cohort was recruited from a tertiary academic center. Lastly, several of our findings trended towards significance, and larger trials are necessary to further elucidate associations with positive CE, complete CE, and hospital readmission.

In conclusion, in this retrospective study of inpatient CE, we found high rates of complete CE, positive CE, and 30-day hospital readmission. Additionally, we correlated CE findings with subsequent procedures. Clinicians may utilize inpatient CE to guide further inpatient management in obscure gastrointestinal bleeding.

## Data Availability

The datasets generated and/or analyzed during the current study are not publicly available due to their containing information such as indirect identifiers that may compromise the privacy of research participants, but are available from the corresponding author on reasonable request.
